# Deep brain stimulation macroelectrodes compared to multiple microelectrodes in rat hippocampus

**DOI:** 10.3389/fneng.2014.00016

**Published:** 2014-06-12

**Authors:** Sharanya Arcot Desai, Claire-Anne Gutekunst, Steve M. Potter, Robert E. Gross

**Affiliations:** ^1^Laboratory for Neuroengineering, Georgia Institute of Technology, AtlantaGA, USA; ^2^The Wallace H. Coulter Department of Biomedical Engineering, Georgia Institute of Technology, AtlantaGA, USA; ^3^Department of Neurosurgery, Emory University School of Medicine, AtlantaGA, USA

**Keywords:** deep brain stimulation, neuronal activation, immediate early gene (IEG), multielectrode array (MEA), macroelectrodes, hippocampus, electroplating

## Abstract

Microelectrode arrays (wire diameter <50 μm) were compared to traditional macroelectrodes for deep brain stimulation (DBS). Understanding the neuronal activation volume may help solve some of the mysteries associated with DBS, e.g., its mechanisms of action. We used c-fos immunohistochemistry to investigate neuronal activation in the rat hippocampus caused by multi-micro- and macroelectrode stimulation. At ± 1V stimulation at 25 Hz, microelectrodes (33 μm diameter) had a radius of activation of 100 μm, which is 50% of that seen with 150 μm diameter macroelectrode stimulation. Macroelectrodes activated about 5.8 times more neurons than a single microelectrode, but displaced ~20 times more neural tissue. The sphere of influence of stimulating electrodes can be significantly increased by reducing their impedance. By ultrasonic electroplating (sonicoplating) the microelectrodes with platinum to increase their surface area and reduce their impedance by an order of magnitude, the radius of activation increased by 50 μm and more than twice the number of neurons were activated within this increased radius compared to unplated microelectrodes. We suggest that a new approach to DBS, one that uses multiple high-surface area microelectrodes, may be more therapeutically effective due to increased neuronal activation.

## INTRODUCTION

While beneficial for neuropsychiatric disorders spanning a wide range (e.g., Parkinson’s disease, depression, epilepsy), much remains to be learned about the mechanisms of action of deep brain stimulation (DBS; Kumar et al., 1998; McIntyre et al., 2004b; Mayberg et al., 2005; Greenberg et al., 2006; Perlmutter and Mink, 2006; Volkmann et al., 2006; Benabid et al., 2009). In particular, a greater understanding the spatial extent of influence of electrical stimulation and the factors that determine it is required to optimize the effectiveness and efficiency of DBS.

All clinical DBS electrodes are single macroelectrodes, cylindrical in shape with diameters ~1 mm and surface areas ~6 mm^2^, although arrays of multiple smaller electrodes have been proposed. However, stimulation through multielectrode arrays (MEA) of microelectrodes (i.e., microstimulation; Desai et al., 2010) may have advantages when compared to single macroelectrode stimulation (i.e., macrostimulation) for several reasons, especially when stimulating complex structures like the hippocampus, an important locus in the etiology of temporal lobe epilepsy (Velasco et al., 2001, 2007). First, microelectrodes with diameters of 10s of microns can be specifically targeted toward particular cell layers, which may not be possible with a macroelectrode whose diameter (e.g., 1.27 mm) is bigger than these targets. Second, one or a few microelectrodes within a MEA may be stimulated at a time, which would enable spatiotemporal patterns of stimulation not possible with a single macroelectrode. Third, tissue damage by MEAs may be less than that caused by macroelectrodes. The first goal of this study was to compare the volume of tissue activated by multimicroelectrode arrays and single macroelectrodes.

Reducing the impedance of microelectrodes provides several advantages for neural recording, such as reduced stimulation artifact and reduced thermal noise leading to improved signal to noise ratio (Ferguson et al., 2009; Desai et al., 2010). For microstimulation, stimulation amplitude is limited to 1 V due to the possibility of water hydrolysis, which therefore limits current density and thus stimulation effectiveness (Rozman et al., 2000). Thus, impedance reduction allows for achieving higher currents in both voltage and current-controlled applications at the near-maximum of safe voltages. A second goal of this study was to compare volume of tissue activated with untreated microelectrodes to that attained in electroplated microelectrodes that are coated to reduce impedance (Desai et al., 2010).

Several simulation studies by Grill (1999), McIntyre et al. (2004a), Wei and Grill (2009), Chaturvedi et al. (2010), Butson et al. (2011), and others have estimated the electrical fields and activating functions surrounding electrode tracks, but this needs validation in living neuronal tissue. An excellent study by Histed et al. (2009) used 2-photon calcium imaging to study patterns of cell activation using microelectrodes in the superficial layers of cortex. Although this technique has great temporal and spatial resolution, it cannot be used in deep brain structures like thalamus or hippocampus because brain tissue strongly scatters light and imaging becomes impossible beyond a depth of 1 mm (Helmchen and Denk, 2005). The results from the Histed et al. (2009) study will certainly serve as a guiding point for understanding electrical stimulation interaction with neuronal populations, but cannot be directly translated to other brain regions which have different cytoarchitecture and projection patterns.

c-fos is an immediate early gene which has been used extensively as a metabolic marker to study seizure pathways and neuroanatomical connections, and to analyze neuronal populations activated by a wide range of stimuli including neuroactive drugs and brain stimulation techniques (Dragunow and Faull, 1989; Saryyeva et al., 2011). Transient induction of c-fos mRNA and protein has been reported in several studies following neuronal excitation (Morgan and Curran, 1991). The mRNA reaches peak values at 30–45 min post-stimulation and decays with a half-life of 12 min. c-fos protein synthesis follows mRNA expression and it is turned over with a half-life of about 2 h (Muller et al., 1984). Given these long activation and decay time scales, this technique lacks temporal resolution. However, it provides single-cell spatial resolution and can be used to visualize and count individual neurons activated by electrical stimulation as shown in Saryyeva et al. (2011) and da Silva et al. (2014). In this study, we used c-fos immunohistochemistry to study neuronal activation caused by DBS in the dorsal hippocampus using macroelectrode, microelectrode, and electroplated microelectrode stimulation.

## MATERIALS AND METHODS

All animal procedures were conducted in accordance with the National Institute of Health Guide for the Care and Use of Laboratory Animals and approved by the Emory University Institutional Animal Care and Use Committee.

**FIGURE 1:**
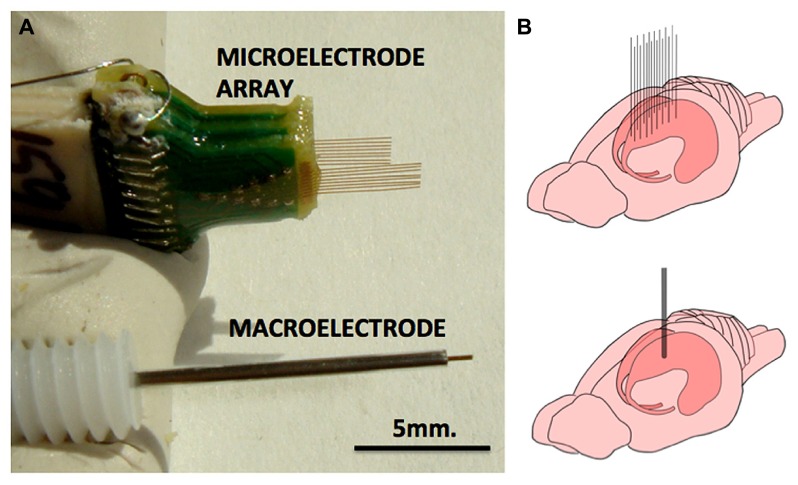
**Macroelectrode and microelectrode array used in this study. (A)** Pictures of microelectrode array (MEA) and macroelectrode of the type that were used in this study. Each of the 16 electrodes of the MEA measure 33 μm in diameter and the macroelectrode measures 150 μm. **(B)** The approximate implantation positions of the MEA and the macroelectrode in the rodent dorsal hippocampus.

**FIGURE 2:**
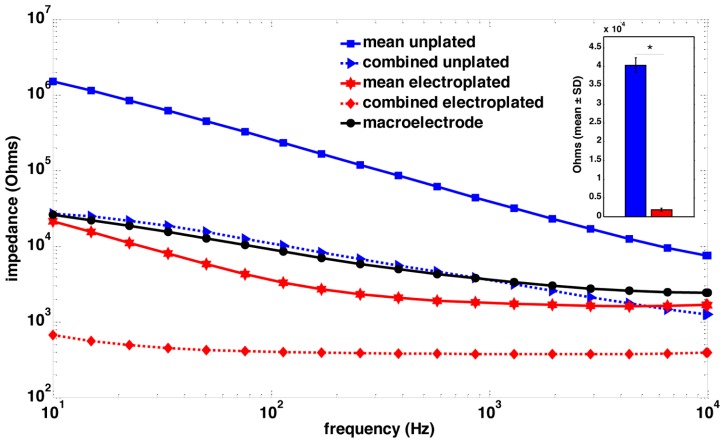
**Impedance spectroscopy of macroelectrode, microelectrode array and sonicoplated microelectrode array.** Impedance spectra of a single macroelectrode (black), the mean impedance of a plated (red) and an unplated (blue) microelectrode array, and the combined impedance of 15 plated (red, dotted) and unplated (blue, dotted) microelectrodes that were connected in parallel for stimulation. The inset shows a bar graph representation of the mean ± SD of the impedance plots at 1 kHz, of the 15 microelecrodes before (blue) and after (red) electroplating. *P < 0.05.

Twenty-four rats were used in this study, divided into three groups of 8. Rats in group 1 had a single macroelectrode (150 μm diameter, Plastics One, VA, USA) aimed toward the CA3 cell layer of the dorsal hippocampus. Rats in groups 2 and 3 were implanted with a microelectrode array (Tucker-Davis Technologies, FL, USA) with 16 33-μm diameter electrodes arranged in two rows of 8 (**Figure 1**). Row 1 was targeted toward the CA1 cell layer and row 2 was targeted toward the CA3 cell layer of the dorsal hippocampus. Whereas group 2 had unplated microelectrodes implanted, the microelectrodes in group 3 had their impedances reduced by an order of magnitude by sonicoplating with platinum black (Desai et al., 2010). Sonicoplating (electroplating under ultrasonic vibrations) significantly improves the durability of platinum black on the base metal. Details on this procedure and its effect on recording and stimulation performance of the microelectrode in chronic implants can be found elsewhere (Desai et al., 2010). Shown in **Figure 2** are impedance plots of a macroelectrode and microelectrode array before and after electroplating. Since microelectrodes in the MEA were connected in parallel while being stimulated, it was necessary to make sure that the combined impedance of 15 microelectrodes (with and without electroplating) did not fall below 200 Ω since the maximum current delivering capacity of our setup is ±5 mA and we used ±1V voltage controlled stimulation in our study.

### SURGERY

Each rat was anesthetized with 1.5–3% inhaled isoflurane before receiving a craniectomy over the right dorsal hippocampus. Each rat was then implanted with either a single macroelectrode (group 1), an unplated microelectrode array (group 2) or a sonicoplated microelectrode array (group 3). Half the rats in each group received 4 h of ±1 V, 400 μs per phase, biphasic square pulses at 25 Hz. Preliminary experiments performed with varying durations of stimulation showed consistently good c-fos expression at 4 h of stimulation. A recent study which analyzed differences between 25 Hz and 130 Hz DBS in the pedunculopontine tegmental nucleus in the rat 6-hydroxydopamine Parkinson’s disease model, also performed 4 h of continuous stimulation (Saryyeva et al., 2011) similar to the methods in the current study. The remaining rats in each group served as controls and did not receive any stimulation. Electrical stimulation was delivered using our custom-built open-source electrophysiology suite, NeuroRighter (Rolston et al., 2009). In cases where the microelectrode array was stimulated, 25 Hz pulses were delivered synchronously through the electrodes. Both the macroelectrode and the MEA electrodes were insulated except at the tip. After 4 h of implantation, electrodes were slowly removed from the brain.

### IMMUNOHISTOCHEMISTRY

Following electrode (macro, micro, or sonicoplated micro) removal from the brain, each rat was deeply anesthetized with a lethal dose of Euthasol (130 mg/kg), injected intraperitoneally, and then perfused intracardially with 0.9% NaCl, followed with 4% paraformaldehyde in 0.1 M phosphate buffered saline at pH 7.2 (PBS) for 15 min at a rate of 20 ml per min. Brains were removed and cryoprotected in 30% sucrose at 4°C, and the region spanning the entire electrode site sectioned in the horizontal plane at 50 μm thickness using a freezing microtome, collected in series of 4 in PBS, and rinsed in PBS. To identify the number and identity of cells activated by the electrical stimulation, double immunofluorescence labeling for the immediate early gene, c-fos (Dragunow and Faull, 1989), and the neuronal marker, NeuN (Mullen et al., 1992) was performed. Free-floating sections were rinsed in PBS, blocked in 5% normal donkey serum (NDS) and 0.1% Triton-X100 for 30 min and rinsed in PBS. After rinses in PBS, sections were incubated overnight at 4°C in rabbit anti-c-fos (1:5000; Calbiochem) and mouse anti-NeuN (1:1000; Millipore) in PBS containing 1% NDS. Sections were rinsed in PBS and incubated in Alexa 594-conjugated donkey anti-rabbit (1:1000; Jackson Immunoresearch) and Alexa 488-conjugated donkey anti-mouse (1:1000; Jackson Immunoresearch) in 1% NDS for 1 h. All sections were additionally counterstained by incubation with 4′,6-diamidino-2-phenylindole (DAPI) that labels all cell nuclei. Sections were rinsed in PBS, and then mounted on glass slides with Fluoromount-G mounting medium (SouthernBiotech) for fluorescence microscopy. For each double-label experiment, controls included omission of one or both primary antibodies. Sections were visualized using a Nikon eclipse E400 microscope equipped with three fluorescent cubes, a monochrome and color digital camera and Nikon BR software (Nikon Instruments Inc., Melville, NY, USA). For each brain at least 2 series were stained and images corresponding to the tip of the electrodes were used for counting.

### CELL COUNTING

In the horizontal sections (50 μm) at the tip of the electrode track, c-fos+/NeuN+ cells surrounding the track were counted using ImageJ and compared between the various stimulation treatments. NeuN and DAPI staining was used to confirm the location of electrodes. Only those electrodes that resided within the hippocampal cell layers were taken into consideration for this study, other electrodes were ignored. Microelectrodes used in this study were separated by 175 μm, but even with careful and slow implantation, they tended to deflect ending closer or farther than 175 μm. Circles in increments of 25 μm in radius were drawn around the electrode tracks in each case and the number of c-fos+/NeuN+ cells were counted within each concentric circle pair in each type of electrode implanted condition (macro, micro, sonicoplated micro; stimulated, unstimulated). With microelectrode array implantation, c-fos+/NeuN+ cells were assigned to the electrode that was closest to the cell. Cell counting was performed until two consecutive circles with no c-fos+/NeuN+ cells were encountered. For computing neuronal activation density, the number of neurons counted within each concentric circle pair was divided by the volume of tissue over which they were counted (*h* = 50 μm for these sections). Test for significance was performed using student’s *t*-test.

## RESULTS

### IMMUNOHISTOCHEMISTRY STAINING OF ELECTRICALLY STIMULATED AND CONTROL SECTIONS

**FIGURE 3:**
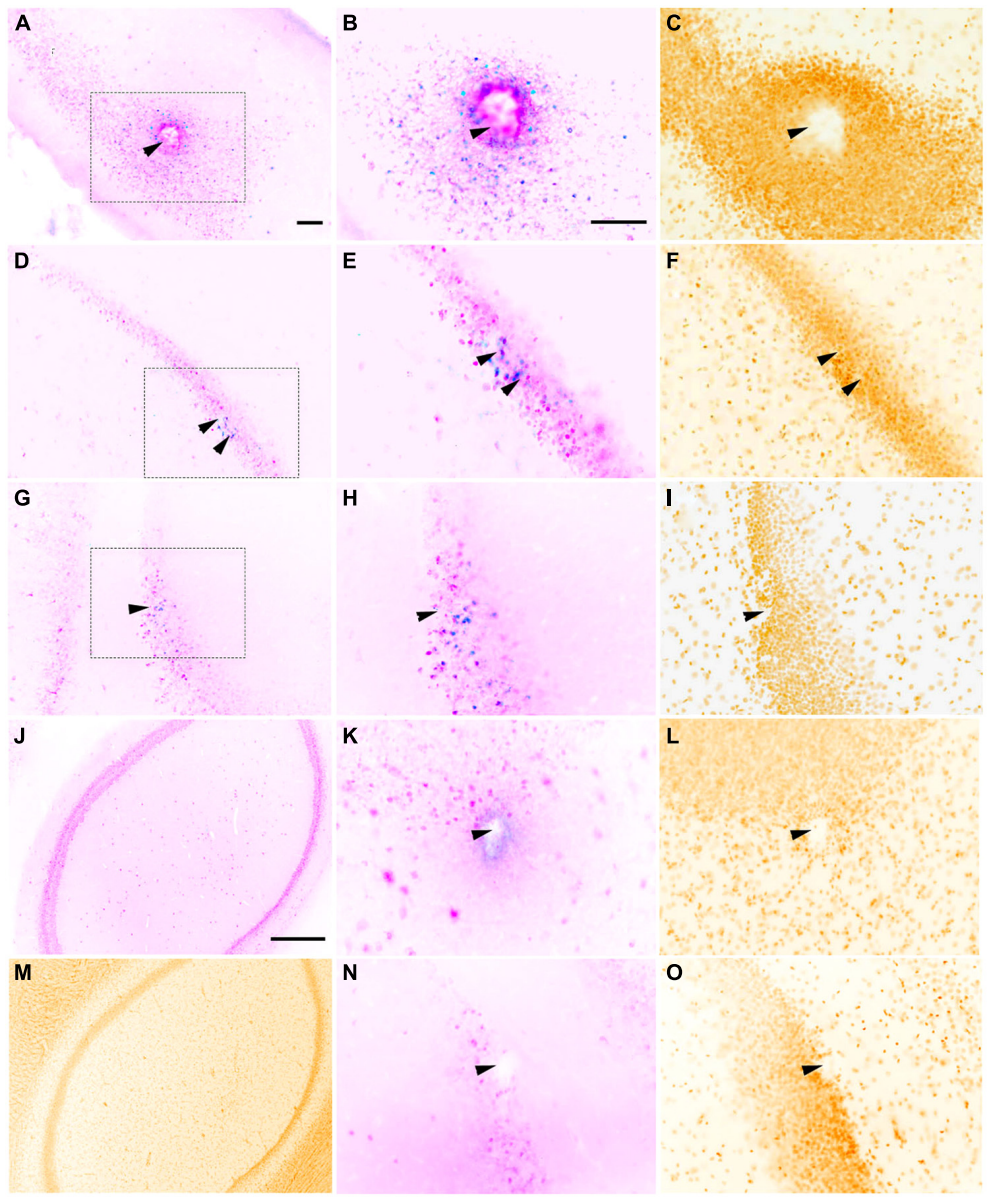
**Representative horizontal sections through the dorsal hippocampus of rats implanted with a macroelectrode (A–C), a microelectrode array (D–F), or a sonicoplated microelectrode array (G–I) stimulated and then stained for c-fos (blue), NeuN (pink) and the nuclear marker DAPI (mustard).** Macroelectrode and microelectrode implanted dorsal hippocampal sections from rats that did not receive any stimulation are shown in **K–L** and **N–O** respectively. **J,M** are sections of the dorsal hippocampus contralateral to the site of macrostimulation. The region within the dotted box in **A,D,** and **G** is shown at higher magnification in **B,E,** and **H**, and **C,F,** and **I**. Location of implanted electrodes is indicated with arrows. Scale bar (for all except **J,M**): 100 μm. **J,M** Scale bar: 500 μm.

Whether the rats were implanted with a macroelectrode, a microelectrode array or a sonicoplated microelectrode array, electrical stimulation resulted in c-fos expression limited to a region immediately surrounding the electrode track, suggesting that electrical stimulation has a limited radius of activation in the dorsal hippocampus (**Figure 3**). Most of the c-fos+ cells were also NeuN+. Local to the site of implantation, c-fos+ and NeuN- cells were also seen in some of the control and stimulated cases. Co-staining for glial cells was not performed, limiting further analysis of these c-fos+/NeuN- cells.

In rats implanted with a macroelectrode or a microelectrode array but not electrically stimulated, no c-fos+/NeuN+ was detected surrounding the electrode tracks (**Figure 3**). These results confirm that it is the electrical stimulation and not the process of electrode implantation itself that is responsible for the increased neuronal c-fos expression seen in the stimulated animals. Dorsal hippocampal sections contralateral to the site of implantation and stimulation also were not c-fos+ in all three cases of stimulation (macro, micro and sonicoplated micro). This suggests that c-fos activation is contained within the hemisphere that received the electrical stimulation.

### NEURONAL ACTIVATION DISTRIBUTION

**FIGURE 4:**
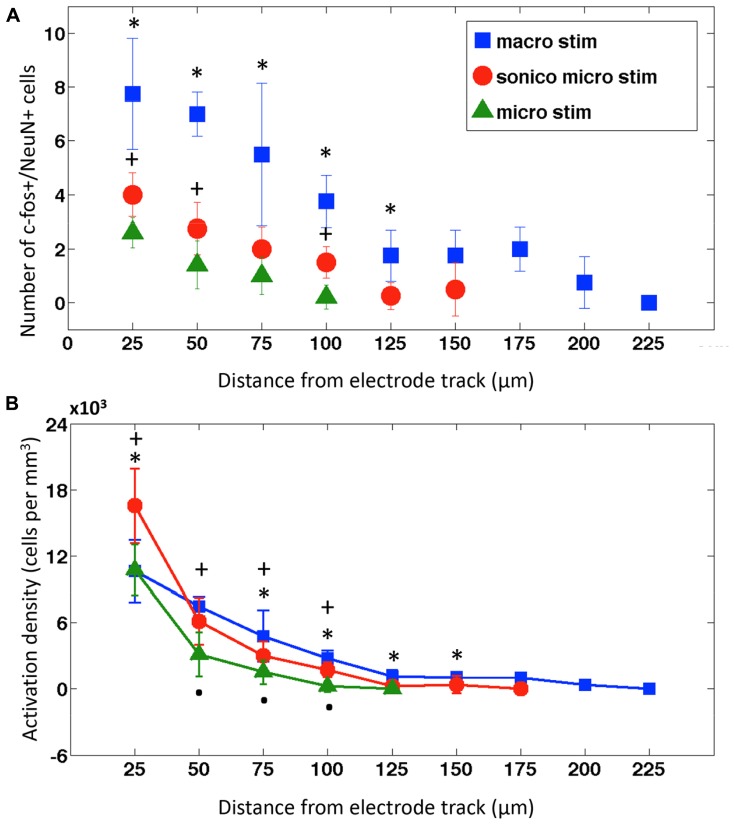
**Neuronal activation distribution around electrode tracks. (A)** Mean ± SD of c-fos expressing neurons within concentric circles in 25 μm radius increments surrounding implanted and stimulated macroelectrode (macro; blue squares, *n* = 4), sonicoplated microelectrode (sonico micro; red circles, *n* = 4) and microelectrode electrodes (micro; green triangles, *n* = 4). *P < 0.05 between macroelectrode, sonicoplated microelectrode, and unplated microelectrode (where data is present). ^+^*P* < 0.05 between sonicoplated and unplated microelectrode. **(B)** Mean ± SD neuronal activation density (number of c-fos+/NeuN+ cells/m^3^) with macroelectrode (macro), sonicoplated microelectrode (sonico micro) and microelectrode (micro) stimulation around the electrode tracks; *P < 0.05 between macroelectrode and sonicoplated microelectrode; ^+^*P* < 0.05 between sonicoplated and unplated microelectrode; *P* < 0.05 between unplated microelectrode and macroelectrode.

As expected, macroelectrodes produced the largest activation radius, followed by sonicoplated microelectrodes and then unplated microelectrodes (**Figure 4A**). When stimulated at 25 Hz, ±1 V, for 4 h, macroelectrodes had a maximum radius of activation of 200 μm and single microelectrodes had radius of activation of 100 μm. Sonicoplated microelectrodes had an increased radius of activation of 150 μm. This was presumably due to their reduced impedance, passing more current at the same fixed voltage. The control cases, where electrodes were implanted but no stimulation was performed, had no c-fos+/NeuN+ cells (**Figure 3**). Interestingly, the sonicoplated microelectrodes had significantly higher activation density (*P* < 0.05) in the first 25 μm concentric cylindrical volume (height of section 50 μm) around the electrode track, when compared to unplated micro- and macroelectrodes (**Figure 4B**). While both microelectrodes had high activation densities in the first concentric cylindrical volume surrounding the electrode track, this rapidly falls with distance, whereas the macroelectrodes had a more linearly decreasing activation density pattern with increasing distance from electrode track.

### NEURONAL ACTIVATION FRACTION AND TOTAL NUMBER OF NEURONS ACTIVATED

**FIGURE 5:**
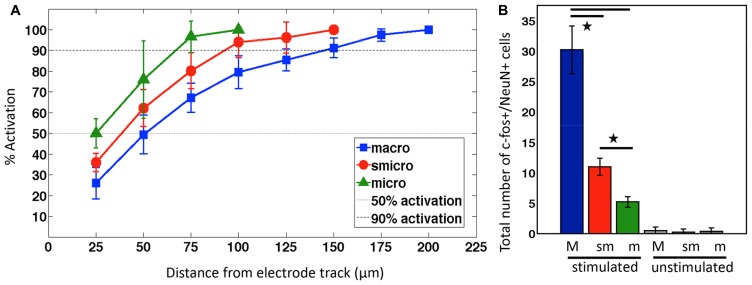
**Neuronal activation density and total number of neurons activated. (A)** Mean ± SD percentage of neuronal activation% in 25 μm radius increments surrounding implanted and stimulated electrodes. **(B)** Bar graphs showing the mean ± SD number of c-fos+/NeuN+ cells with macroelectrode (M), sonicoplated microelectrode (sm), or unplated microelectrode (m), with and without stimulation. *P < 0.05.

The cumulative activation **Figure 5A** shows that with macroelectrode stimulation, 50% of activated neurons (AN_50_) were within a radius of 50.7 μm from the electrode track and 90% (AN_90_) were within a radius of 144.7 μm. At the other extreme, sonicoplated microelectrodes AN_50_ was 38.3 μm AN_90_ was 92.7 μm, with intermediate values for the unplated microelectrode (AN_50_, 25 μm; AN_90_, 70 μm). A single macroelectrode activated 30.25 ± 3.95 neurons surrounding the electrode track in the dorsal hippocampus, in one 50 μm brain section compared to 5.2 ± 0.84 neurons with a single microelectrode. Sonicoplating resulted in a 52.7% increase in the number of neurons activated (11 ± 1.41). In all the unstimulated controls, the number of c-fos+/NeuN+ was not statistically different from background c-fos activation levels (**Figure 5B**)

### NEURONAL ACTIVATION WITH MICROELECTRODE ARRAYS

**FIGURE 6:**
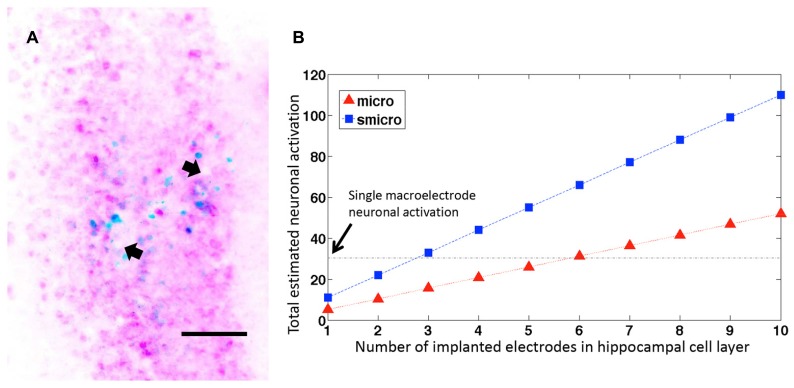
**Neuronal activation with several microelectrodes. (A)** Representative horizontal section through the dorsal hippocampus of a rat implanted with a microelectrode array. The image shows two microelectrode sites (depicted with arrows) ending within the hippocampal pyramidal cell layer. The section is stained for c-fos (blue) and NeuN (pink). Scale bar: 100 μm. **(B)** Estimated neuronal activation in 50 μm brain sections with multiple unplated and sonicoplated microelectrodes (micro, smicro respectively) implanted in the hippocampal cell layer. The horizontal black dotted line shows the mean number of neurons activated by a single macroelectrode for comparison.

In cases where more than one microelectrode from the implanted microelectrode array ended within the pyramidal cell layer of dorsal hippocampus, the number of c-fos+/NeuN+ cells increased linearly with the number of electrodes within the cell layer (**Figure 6A**). If we extrapolate these results (**Figure 6B**), it would take fewer than 6 unplated microelectrodes (at 33 μm diameter they would occupy 28% volume compared to a macroelectrode) or fewer than 3 sonicoplated microelectrodes (13.3% volume compared to macroelectrode) to activate an equal number of neurons as a single macroelectrode (150 μm diameter). Alternatively, with 20 unplated or sonicoplated microelectrodes (which would occupy less volume compared to a single macroelectrode), 3.5 or 7.5 times more neurons would be activated when compared to a single macroelectrode.

## DISCUSSION

Using c-fos immunohistochemistry, we have compared the radius of activation and the activation density following electrical stimulation through a macroelectrode, a microelectrode array and an electroplated microelectrode array in the dorsal hippocampus. A macroelectrode has an activation radius of 200 μm, a microelectrode has an activation radius of 100 μm and an electroplated microelectrode has an activation radius of 150 μm. Thus, a microelectrode array approach could be used when precise stimulation of narrow cell layers, such as the pyramidal cells layer of the CA1 and CA3 regions of the hippocampus is desired. By targeting unplated or electroplated microelectrodes to certain cell layers, stimulation of structures outside these targets can be minimized. The activation radius numbers presented here would help in determining the spacing between microelectrodes in the microelectrode array. One method of achieving precise targeting of microelectrodes to specific cell layers would be through the use of movable microelectrodes (Rolston et al., 2011) whose depth can be individually adjusted using microdrives (Keating and Gerstein, 2002). Using a macroelectrode whose diameter is larger than these targets would lead to stimulation of structures outside the target, giving rise to undesired side effects (Hariz, 2002).

The macroelectrode and the microelectrode array used in this study are from different manufacturers and are made of different material. The macroelectrode was made of stainless steel with polyimide insulation, whereas the microelectrodes were made of tungsten with polyimide insulation and the electroplated material was platinum black. These materials have different charge storage capacities (Merrill et al., 2005) and hence may employ different degrees of capacitive vs. faradaic charge transfer mechanisms (Merrill et al., 2005; Cogan, 2008). Therefore, the differences seen here between the three stimulation cases may arise not only due to the differences in their geometry, but also due to differences in their material composition. We note here that it is common for macroelectrodes to be built from material such as the stainless steel (Gimsa et al., 2005) that have lower charge storage capacity. Metals such as tungsten, platinum or platinum-iridium have higher charge storage capacities and are the popular choice for making microelectrodes (Stoney et al., 1968). The larger size of the macroelectrode gives it sufficient surface area to inject large currents safely (Mccreery et al., 1990). While we acknowledge that a direct comparison of the effects of increasing only the size of electrodes on the activation radius would require the electrodes to be made of the same material, a study of that nature would have less practical relevance since materials such as platinum-iridium are rarely used to make the macroelectrodes used in animal studies.

Only one electrical stimulation parameter (±1 V, 400 μs/phase, biphasic square pulses at 25 Hz) was tested in this paper. Since different populations of neurons within the hippocampus have a preference to fire when stimulated at distinct frequencies (For example Pike et al., 2000 shows that pyramidal cells have a lower frequency preference while fast spiking interneurons prefer higher frequencies), we anticipate that stimulating at higher/lower frequencies and voltages will excite different populations and numbers of neurons (Wagenaar et al., 2004). For example, a study performed in the pedunculopontine nucleus in the rat 6-hydroxydopamine (6-OHDA) Parkinson’s disease model using c-fos immunohistochemistry showed that 25 Hz stimulation activated more neurons when compared to 130 Hz stimulation (Saryyeva et al., 2011).

In chronic implantation studies, glial encapsulation of the implanted electrode increases the distance of the target neurons from the implanted electrode (Griffith and Humphrey, 2006; Bewernick et al., 2010). Although we did not look into the effects of gliosis on the number of neurons activated, we hypothesize that this process would cause a steady decrease in neuronal activation over time in voltage controlled stimulation studies due to increased impedance of the tissue-electrode interface (Butson et al., 2006). Additional empirical studies should address the effects of glial encapsulation on neuronal activation. In chronic implantation experiments, changes in impedance over time should be closely monitored and stimulation parameters should be adjusted to account for this increased impedance to achieve a better control on neural activation, although this is compensated directly in current-controlled approaches. Using a closed-loop electrophysiology setup [e.g., NeuroRighter (Newman et al., 2012)] will greatly help in automating this process.

As discussed above (see Results), the implantation of the macroelectrode used in this study (150 μm diameter) would cause 20.67 times the tissue damage of implantation of a single microelectrode (33 μm diameter). For human DBS electrodes, which are typically 1.27 mm in diameter, this ratio would be much larger. By positioning microelectrodes sufficiently close within a given target, more neurons can be activated than using a single macroelectrode while causing less tissue damage.

## Conflict of Interest Statement

The authors declare that the research was conducted in the absence of any commercial or financial relationships that could be construed as a potential conflict of interest.
